# Selective Recruitment of a Synthetic Histone Acetyltransferase Can Boost CHO Cell Productivity

**DOI:** 10.1002/biot.202400474

**Published:** 2024-12-10

**Authors:** Sienna P. Butterfield, Rebecca E. Sizer, Fay L. Saunders, Robert J. White

**Affiliations:** ^1^ Department of Biology University of York York UK; ^2^ FUJIFILM Diosynth Biotechnologies Billingham UK

**Keywords:** CHO, DNA methylation, epigenetics, histone acetylation, p300

## Abstract

Industrial production of biologics typically involves the integration of transgenes into host cell genomes, most often Chinese hamster ovary (CHO) cells. Epigenetic control of transgene expression is a major determinant of production titers. Although the cytomegalovirus (CMV) promoter has long been used to drive industrial transgene expression, we found that its associated histones are suboptimally acetylated in CHO cells, providing an opportunity to enhance productivity through epigenetic manipulation. Expression of monoclonal antibody mRNAs increased up to 12‐fold when a CRISPR‐dCas9 system delivered the catalytic domain of a histone acetyltransferase to the CMV promoter. This effect was far stronger than when promoter DNA was selectively demethylated using dCas9 fused to a 5‐methylcytosine dioxygenase. Mechanistically, acetylation‐mediated transcriptional activation involved heightened phosphorylation and activity of RNA polymerase II, enabling it to escape promoter‐proximal pausing at the transgene. This approach almost doubled the titer and specific productivity of antibody‐producing CHO cells, demonstrating the potential for biomanufacturing.

## Graphical Abstract and Lay Summary

Epigenetic control of transgene expression is a major determinant of production titers. Expression of monoclonal antibody mRNAs increased substantially when a CRISPR‐dCas9 system delivered the catalytic domain of a histone acetyltransferase to transgene promoters. This significantly improved the titer and specific productivity of CHO cells producing a monoclonal antibody, demonstrating potential for biomanufacturing.

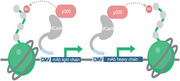

## 1 Introduction

CHO cells are established platforms for industrial manufacture of biological therapeutics, such as mAbs [1, 2]. However, a major challenge is the instability of transgene expression over long‐term culture, which necessitates costly stability studies [3]. This instability can arise through copy number loss, genomic rearrangements, or epigenetic silencing of the transgene [4, 5, 6]. Following random integration into the CHO cell genome, the transgene and its promoter are subject to epigenetic influences that can suppress expression immediately or gradually [7, 8, 9, 10].

The epigenome comprises covalent modifications of histones and DNA that can recruit transcription regulators and alter accessibility [7, 8, 9, 10]. Heterochromatin is inaccessible to most transcription factors and is associated with silenced genes. It is demarcated with highly methylated DNA, histone hypoacetylation, and trimethylation of lysines 9 and 27 of histone H3 (H3K9me3 and H3K27me3, respectively) [7, 11, 12]. Accessible regions with active transcription are termed euchromatin and feature histone hyperacetylation, particularly of lysines 9 and 27 of histone H3 (H3K9ac and H3K27ac, respectively) [12, 13, 14]. Indeed, enrichment of H3K27ac is a key criterion in defining active promoters [12, 13, 14].

Histone marks and DNA methylation shift significantly during protracted culture of CHO cells, alongside major changes in the transcriptome and phenotypic drift [5, 6, 7, 8, 15, 16, 17]. The epigenetic state of promoters is especially significant in determining transcription potential [15]. The human cytomegalovirus (CMV) promoter has long been used for driving high transgene expression in mammalian cells [18]. Despite extensive sequence optimization and identification of key functional regulators of the CMV promoter, its activity falls during extended culture [18, 19, 20]. DNA methylation of a specific CpG is associated with production instability and hypoacetylation of histone H3 may accompany loss of productivity in unstable CHO clones [10, 20, 21, 22, 23, 24]. Conversely, H3 hyperacetylation on the CMV promoter correlates with production stability [25].

Barriers can protect the transgene and promoter to maintain expression stability and combat epigenetic silencing. Ubiquitous chromatin opening elements (UCOEs), tRNA genes and matrix attachment regions (MARs) are examples of such elements with barrier and/or transcription‐boosting abilities [26, 27, 28, 29, 30]. Modifying the activities of epigenetic modifiers, such as histone deacetylases (HDACs) and histone acetyltransferases (HATs), provides opportunities to manipulate the epigenetic landscape. Global HDAC inhibition using small molecule drugs like sodium butyrate can induce histone hyperacetylation at transgene promoters and increase the expression of transgenes and endogenous genes in CHO cells [11, 31]. However, these drugs are unsuitable for industrial biological production because of the pleiotropic effects of genome‐wide histone hyperacetylation, which can impact multiple activities, including proliferation and viability. Changes in histone methylation also occur as an indirect consequence of HDAC inhibitors [32]. Furthermore, they can impact product quality and require removal during downstream processing [11, 31].

Targeted epigenetic editing is a desirable alternative, which can be achieved using CRISPR technology. A dead version of Cas9 (dCas9), containing mutations in both nuclease domains, can target specific sites without cleaving DNA and can be fused to effector domains from epigenetic modifiers, to deliver these specifically to transgenes and manipulate their expression selectively [33, 34, 35, 36, 37, 38]. For example, targeting the HAT p300 to specific promoters by CRISPR‐dCas9 boosted locus‐specific H3K27ac and transcriptional output [36, 39].

This study describes the epigenetic manipulation of the CMV promoter. Although it is a powerful expression system, we found suboptimal histone acetylation at the CMV promoter in CHO cells. Selectively increasing H3K27ac with p300‐dCas9 significantly increased mAb transgene expression and fully restored productivity lost during culture. Targeting the DNA demethylase TET1 to the promoter with dCas9 reversed DNA methylation and partially alleviated silencing. This programmable epigenome editing can ameliorate silencing and raise biological production in CHO cells.

## 2 Materials and Methods

### 2.1 Generation of dCas9‐Expressing Cells

#### 2.1.1 Single Guide RNA Design and Plasmid Preparation

Four single‐guide RNAs (sgRNAs 1–4) targeting the CMV promoter were designed with the Benchling guide RNA tool (https://benchling.com) (Table 1) and cloned into the pSpgRNA plasmid (Addgene 47108), as previously described [37, 40]. Plasmids pAW91‐dCas9 (Addgene 104372), pcDNA‐dCas9‐p300 Core (Addgene 61357), p‐dCas9‐HDAC1‐Hygro (Addgene 104409), Fuw‐dCas9‐Tet1CD‐P2A‐BFP (Addgene 108245) (termed dCas9, dCas9‐p300, dCas9‐HDAC1, and dCas9‐TET1, respectively) and sgRNAs 1–4 were amplified in *Escherichia coli* cultures in Luria‐Bertani broth, isolated with QIAGEN Plasmid Midi Kit and eluted in RNase‐free distilled water.

**TABLE 1:** Guide RNA sequences.

sgRNA name	Sequence (5’‐3’)
*sgRNA 1 F*	CACCGCCTGGCATTATGCCCAGTAC
*sgRNA 1 R*	AAACGTACTGGGCATAATGCCAGGC
*sgRNA 2 F*	CACCGATTAGTCATCGCTATTACCA
*sgRNA 2 R*	AAACTGGTAATAGCGATGACTAATC
*sgRNA 3 F*	CACCGACATCAATGGGCGTGGATAG
*sgRNA 3 R*	AAACCTATCCACGCCCATTGATGTC
*sgRNA 4 F*	CACCGCCCCATTGACGCAAATGGG
*sgRNA 4 R*	AAACCCCATTTGCGTCAATGGGGC

Abbreviations: F = forward sequence, R = reverse sequence.

#### 2.1.2 Generating eGFP and mAb‐Expressing Cell Pools

**FIGURE 1:**
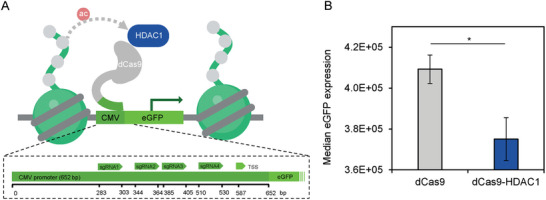
Targeting HDAC1 to the CMV promoter reduces expression of eGFP. (A) Schematic depicting a pool of 4 sgRNAs targeting dCas9‐HDAC1 to the CMV promoter causing deacetylation of histone tails. (B) Median eGFP fluorescence of CHO‐K1 cells stably expressing dCas9 and dCas9‐HDAC1, as measured by flow cytometry. *p < 0.05 (*t*‐test), error bars represent ± SEM, *n = 3*.

**FIGURE 2:**
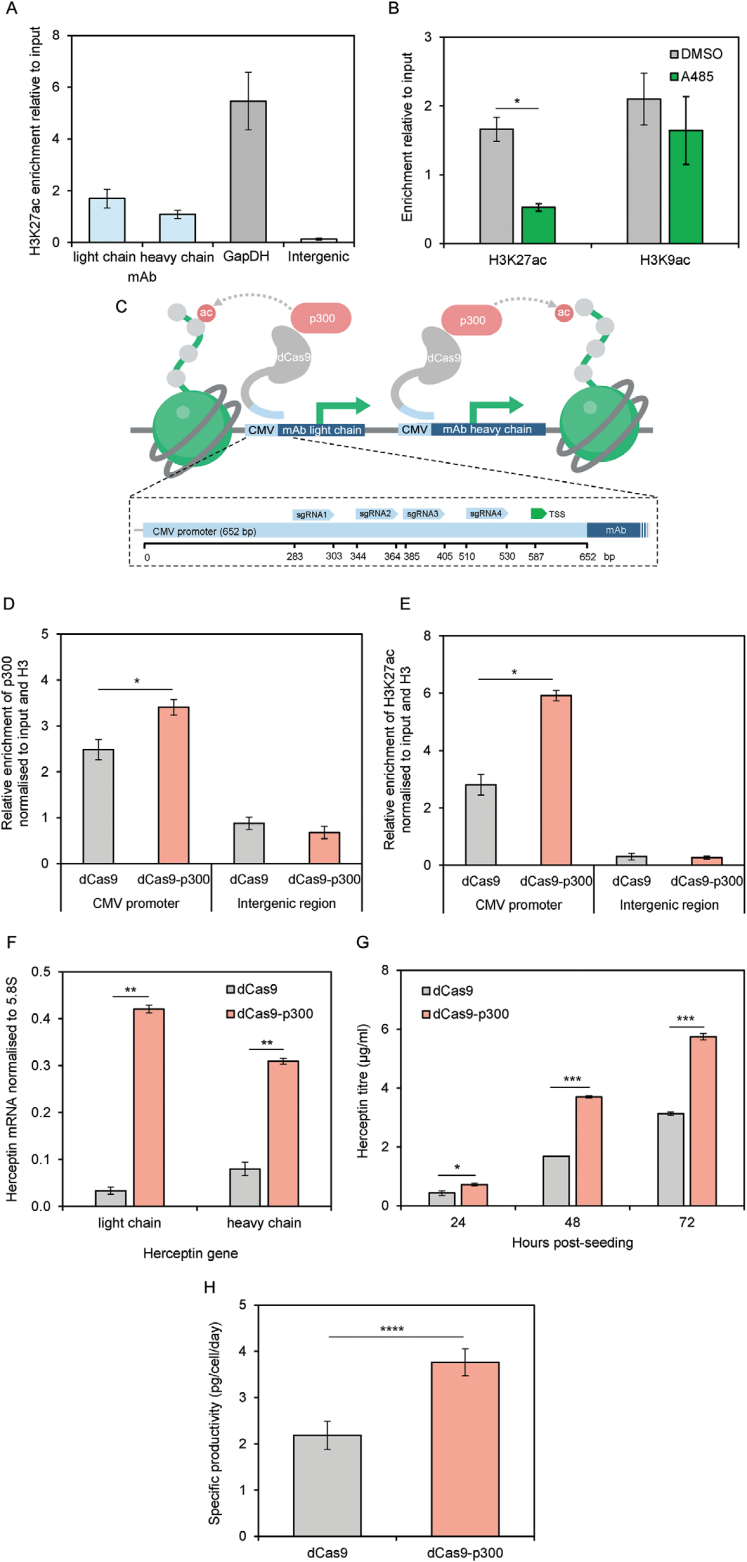
Recruitment of p300 using CRISPR/dCas9 can increase locus‐specific histone acetylation, expression, and antibody production. (A) ChIP data showing enrichment of H3K27ac at the CMV promoter driving light and heavy chain mAb genes and the endogenous GapDH promoter in CHO‐K1 cells. (B) ChIP data showing enrichment of H3K27ac and H3K9ac at the CMV promoter in CHO‐K1 cells following A‐485 treatment. (C) Schematic depicting pooled sgRNAs targeting the dCas9‐p300 fusion to the CMV promoter driving mAb light and heavy chain genes in CHO‐K1 cells. (D–E) ChIP data showing enrichment of p300 (D) and H3K27ac (E) at the CMV promoter and an intergenic region, normalized to input and total H3. (F) mAb light and heavy chain mRNA levels normalized to 5.8S rRNA, as measured by RT‐qPCR in CHO‐K1 cells stably expressing dCas9‐p300 or dCas9. (G) mAb titers of cell pools stably expressing dCas9‐p300 compared to dCas9 alone. (H) Cell‐specific productivities (q_p_s) calculated using titer values. *p < 0.05, **p < 0.01, ***p < 0.001, ****p < 0.0001, n.s. (not significant) (*t*‐test). Error bars represent ± SEM, *n = 3*.

CHO‐K1 cells (ATCC) were transfected with constructs expressing eGFP or mAb heavy and light chain genes driven by a CMV promoter. Transfection used 5 µg DNA in a volume of 100 µL and followed the Xfect protocol (TakaraBio) for 6‐well plates (*n* = 3). For selection, media was supplemented 48 h post‐transfection with 5 µg/mL puromycin every 2–3 days for 2 weeks to generate populations of CHO‐K1‐eGFP cells (Figure 1) and CHO‐K1‐mAb cells (Figure 2). Routine culture was in Ham's F12 medium with 10% fetal calf serum, 4 mM GlutaMAX (Sigma Aldrich), and 1% penicillin/streptomycin in T flasks at 37°C with 5.4% CO_2_.

#### 2.1.3 Generating dCas9‐Expressing Cell Pools

For transfection into early passage CHO‐K1‐eGFP cell pools, dCas9 and sgRNA plasmids 1–4 were mixed in a molar ratio of 1:1:1:1:1 (dCas9:sgRNA1:sgRNA2:sgRNA3:sgRNA4) with 5 µg total DNA in 100 µL, following the Xfect protocol (TakaraBio) for 6‐well plates (*n* = 3). This was repeated for dCas9‐HDAC1. Early passage CHO‐K1‐mAb cell pools were transfected with dCas9 and dCas9‐p300 with sgRNAs 1–4. For selection, media was supplemented 48‐h post‐transfection with 250 µg/mL Zeocin (for dCas9), 400 µg/mL G418 (for dCas9‐p300) or 100 µg/mL Hygromycin (for dCas9‐HDAC1) every 2–3 days for 2 weeks.

#### 2.1.4 Generating dCas9‐Expressing Cells for Stability Studies

A CHO‐K1‐mAb clonal cell line was isolated and cultured for 10 weeks. Samples were cryopreserved in FBS with 10% DMSO at weeks 0 and 10. After simultaneous revival, week 0 and week 10 cells were transfected with sgRNAs and dCas9, dCas9‐TET1, dCas9‐p300 or both dCas9‐p300 and dCas9‐TET1, as above (*n* = 3). For selection, media was supplemented 48 h post‐transfection with 250 µg/mL Zeocin (for dCas9 and dCas9‐TET1) or 400 µg/mL G418 (for dCas9‐p300) every 2–3 days for 2 weeks. Simultaneous selection of dCas9‐TET1 and dCas9‐p300 required 250 µg/mL Zeocin with 400 µg/mL G418.

### 2.2 Flow Cytometry

Flow cytometry analysis for eGFP reporter assays used an LX375 CytoFLEX flow cytometer (Beckman Coulter). Approximately 0.25 × 10^6^ cells were resuspended in 250 µL media. Non‐fluorescing CHO‐K1 cells were used to establish a gating strategy and to set the background fluorescence threshold. Forward scatter (FSC) and side scatter (SSC) settings were adjusted accordingly to differentiate live cells from debris. The eGFP‐positive population was identified using the B525‐FITC‐A channel (488 nm excitation, 525/40 nm emission). Acquisition settings were adjusted to separate eGFP‐positive and negative cells. The analysis utilized CytExpert software (Beckman Coulter). Flow cytometry was repeated using cell pools from three biological repeats. At least 10,000 events were collected per sample.

### 2.3 ChIP‐qPCR

Chromatin was extracted using a method adapted from Ghisletti et al. [41]. 0.5 × 10^6^ CHO‐K1‐mAb cells were fixed with 16% formaldehyde, lysed and then sonicated with the BioruptorPlus (Diagenode) using 10 cycles of [30 s “ON”, 30 s “OFF”]. Immunoprecipitation used 5 µg per ChIP of antibodies: H3K27ac (Abcam, ab4729); p300/KAT3B (Abcam, ab275378); H3K9ac (Abcam, ab4441); Pol II (Abcam, ab52202); TBP (Abcam, ab818); Pol II S2P (Abcam, ab193468); BRD4 (Fisher Scientific, 15907532); CDK9 (Abcam, ab239364); H3 (Abcam, ab1791) and pre‐immune serum negative control, produced in‐house. ChIP‐qPCR primers are shown in Table 2. ChIP experiments were repeated using cell pools from three biological repeats. Each biological repeat sample was loaded in duplicate on an Admiral cycler with 45 amplification cycles. Enrichment was calculated in Excel and normalized to 1% of the starting chromatin (input). Treatment with A485 was observed to reduce cell proliferation, but normalization of ChIP data to amounts of chromatin compensates for differences in cell number.

**TABLE 2:** ChIP‐qPCR and RT‐qPCR primer sequences.

Target gene/region	Sequence (5’‐3’)
*CMV promoter F (ChIP)*	ATGTCGTAACAACTCCCCCC
*CMV promoter R (ChIP)*	TAGCGGATCTGACGGTTCAC
*GapDH promoter F (ChIP)*	TTCTAGAGACAGCCGCATCTT
*GapDH promoter R (ChIP)*	CCCCGTTTCCGACCGT
*Intergenic region F (ChIP)*	CAAGTTCATCTGCGTCCAA
*Intergenic region R (ChIP)*	GCTACTTTGTCTGGGGAACA
*5.8S F (RT‐qPCR)*	TCGATGAAGAACGCAGCTA
*5.8S R (RT‐qPCR)*	GTGCGTTCGAAGTGTCGA
*mAb light chain (LC) F (RT‐qPCR)*	AGTCCCTTCTCGCTTCTCTG
*mAb light chain (LC) R (RT‐qPCR)*	CTCCACCTTGGTACCCTGTC
*mAb heavy chain (HC) F (RT‐qPCR)*	AGTTCAACTGGTACGTGGAC
*mAb heavy chain (HC) R (RT‐qPCR)*	TACGTGCTGTTGTACTGCTC

Abbreviations: F = forward sequence, R = reverse sequence.

### 2.4 mRNA Expression by RT‐qPCR

Total RNA from approximately 1 × 10^6^ cells was isolated from stably‐expressing cell pools using TRIzol reagent (Invitrogen). Concentration and quality of RNA were measured on a Nanodrop One (Thermo Fisher Scientific). cDNA was produced using the SuperScript IV Low‐Input cDNA PreAmp kit (Thermo Fisher Scientific) with RNasin Plus inhibitor (Promega) in 20 µL reactions. cDNA samples alongside RNA samples as negative controls were used as templates in a 20 µL reaction with primers against target genes (Table 2). RT‐qPCR experiments were repeated using cell pools from three biological repeats. Each biological repeat sample was then loaded in duplicate on an Admiral cycler with 45 amplification cycles. Relative transcript levels were normalized to 5.8S rRNA using the 2^−∆∆CT^ method.

### 2.5 ELISA

Production of mAb was quantified by ELISA. Cell pools were seeded at 0.3 × 10^6^ per well in 3 mL media in 6‐well plates. Samples of culture medium were collected each day and stored at ‐80°C after removing cells by centrifugation. 96‐well plates coated with Protein A were washed (PBS, 0.05% Tween20). Media samples and purified human IgG were serially diluted (PBS, 0.05% Tween20), added in triplicate to the 96‐well plate, and incubated for 1 h at room temperature. Samples were discarded and plates were washed (PBS, 0.05% Tween20). Chicken pAb to human IgG HRP (Abcam, ab112454) in Superblock blocking buffer was added to wells and incubated for 1 h at room temperature. After washing, SuperSignal ELISA Pico (Thermo Fisher Scientific, 1859677) detection agent was added and mAb levels were quantified on a CLARIOstar Plate Reader (BMG Labtech).

### 2.6 CpG Methylation

For DNA methylation analysis, the EpiMark Methylated DNA Enrichment Kit (NEB, E2600S) was used. 5 mg genomic DNA was extracted using the Monarch Genomic DNA Purification Kit (New England BioLabs) and sonicated to 250 bp using a Focused Ultrasonicator (Covaris). Methylated DNA was isolated using Protein A magnetic beads coupled to MBD2‐Fc protein. Following incubation, unbound DNA was washed off, and captured methylated CpG DNA was eluted in DNase‐free water. The resulting enriched methyl CpG‐containing DNA was subjected to qPCR carried out with ChIP‐suitable primers against target regions (Table 2). Samples from three biological repeats were measured in duplicate on the Admiral cycler with 45 amplification cycles. Enrichment was calculated in Excel and values were normalized to 1% of the starting material (input).

## 3 Results

### 3.1 Deacetylation of Histones at the CMV Promoter Lowers Expression

CHO‐K1 cells were stably transfected with an eGFP reporter gene driven by the CMV promoter. A pool of four sgRNAs targeting this promoter was then used to direct specific binding of dCas9‐HDAC1, where catalytically‐inactive Cas9 is fused to the histone deacetylase HDAC1 (Figure 1A). Targeting dCas9 alone to a promoter can reduce transcription by sterically hindering the binding and progression of RNA polymerase [33]. Nevertheless, when the dCas9‐HDAC1 fusion was targeted to the CMV promoter, median eGFP expression was significantly reduced (*p* < 0.05) relative to expression with dCas9, as measured by flow cytometry (Figure 1B). These data provide evidence that localized acetylation at the CMV promoter can influence transgene output significantly.

### 3.2 Sub‐Optimal Acetylation of Nucleosomes at the CMV Promoter

Histone acetylation correlates strongly with promoter activity [12, 13, 14]. Using ChIP‐qPCR, we compared H3K27ac at the CMV promoter driving stable expression of mAb transgenes with that of the strong endogenous promoter of the glyceraldehyde 3‐phosphate dehydrogenase (GAPDH) gene, which is expressed at high levels [42]. Relative enrichment of H3K27ac was 3.2‐fold and 5.0‐fold lower at the CMV promoter of the mAb light and heavy chain genes, respectively, compared to the GAPDH promoter (Figure 2A). Thus, at least some copies of the CMV promoter have suboptimal acetylation of H3K27 in pools of cells with randomly integrated vectors. This raises the possibility of enhancing transgene expression by increasing acetylation of local histones.

#### 3.2.1 Locus‐Specific Histone Acetylation at the CMV Promoter Can Be Achieved Using dCas9‐p300

In other cell types, H3K27 is acetylated by the HAT p300/KAT3B and its homologue, CBP/KAT3A [43]. We used A‐485, a highly specific inhibitor of p300 and CBP [43], to test if these enzymes are responsible for acetylating H3K27 on the CMV promoter driving mAb transgenes in CHO‐K1 cells. ChIP‐qPCR revealed that A‐485 caused a significant (*p* < 0.05) 3.2‐fold decrease in H3K27ac at the CMV promoter, whereas H3K9ac did not change significantly (*p* > 0.05) (Figure 2B). This is consistent with p300 and/or CBP contributing selectively to H3K27 acetylation in CHO‐K1 cells, as in other cell types.

CHO‐K1 cells were created that stably express dCas9 fused to the core HAT domain of p300 (dCas9‐p300) and sgRNAs1‐4 that target the CMV promoter (Figure 2C). Cells expressing dCas9‐p300 proliferated at similar rates to those expressing dCas9. ChIP‐qPCR demonstrated significant enrichment of p300 (1.4‐fold, *p* < 0.05) and H3K27ac (2.1‐fold, *p* < 0.05) at the CMV promoter in cells expressing dCas9‐p300, relative to those expressing unfused dCas9. Specificity was confirmed by the absence of enrichment at an untargeted control intergenic region (Figure 2D–E) [36]. These results confirm that specific targeting of the p300 HAT region can increase H3K27ac at the CMV promoter.

### 3.3 Recruiting p300 to the CMV Promoter Increases mAb Production

To investigate whether p300 recruitment to CMV promoters impacts transgene expression, mAb mRNA transcripts were quantified by RT‐qPCR in CHO‐K1 cells stably expressing dCas9 or dCas9‐p300. MAb light and heavy chain mRNAs were significantly (*p* < 0.01) elevated in cells expressing dCas9‐p300, by 12.5‐fold and 3.9‐fold respectively, compared to cells expressing dCas9 alone (Figure 2F). This is consistent with strong positive correlations between promoter H3K27ac and transcription [36].

The increase in mAb mRNA was sufficient to raise the titer of the secreted antibody. In CHO‐K1 cells with dCas9‐p300 targeted to the CMV promoter, mAb titer was 1.8‐fold higher than in cells expressing dCas9, 72 h after seeding (Figure 2G). This corresponded to 2.6 µg/mL (*p* < 0.001) more mAb production and 1.7‐fold higher specific productivity (*p* < 0.0001) (Figure 2H). Targeting the catalytic domain of p300 to the CMV promoter can therefore increase H3K27 acetylation, mAb mRNA, titer, and specific productivity.

### 3.4 Demethylation of DNA at the CMV Promoter Increased Transcription but Was Less Effective Than H3K27 Acetylation

**FIGURE 3:**
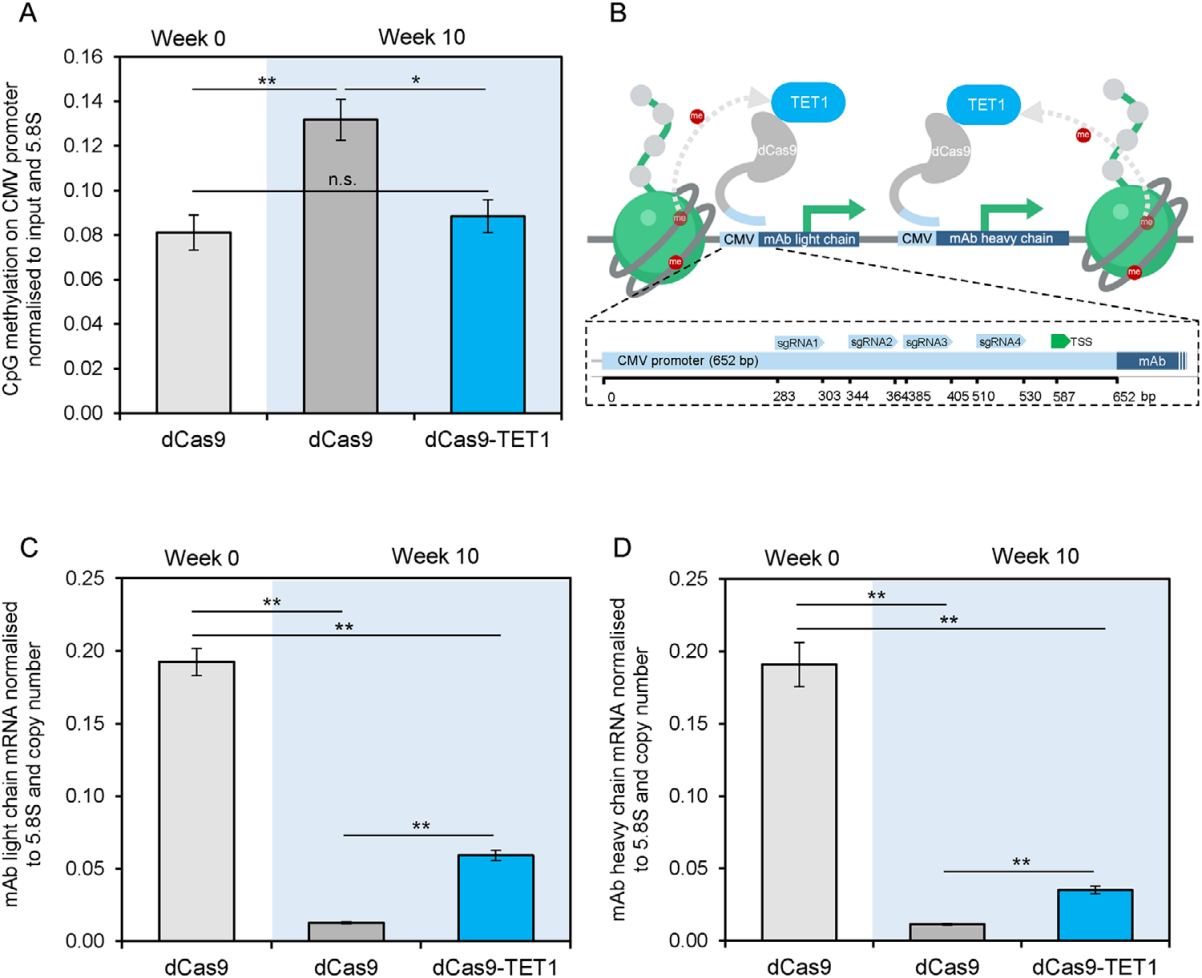
Demethylation of a transgene promoter by targeting TET1 can increase transcription. (A) CpG methylation at the CMV promoter in a CHO‐K1 cell line was measured at week 0 and week 10 of culture. Targeting the DNA demethylase TET1 to the CMV promoter reversed the CpG methylation that had been gained from week 0 to week 10. Enrichment is normalized to input. (B) Schematic depicting a pool of 4 sgRNAs targeting the dCas9‐TET1 fusion protein to the CMV promoter driving mAb light and heavy chain genes in CHO‐K1 cells. (C,D) Corresponding mAb light chain (C) and heavy chain (D) mRNA expression levels in the same CHO‐K1 cell line were measured at week 0 and week 10 of culture expressing either dCas9 alone or dCas9‐TET1. mRNA levels are normalized to 5.8S rRNA and copy number. *p < 0.05, **p < 0.01, n.s. not significant (test). Error bars represent ± SEM, *n* = 3.

DNA methylation has been implicated in transgene silencing in CHO cells [11, 37]. Consistent with this, we found a highly significant (*p* < 0.01) increase in DNA methylation at the CMV promoter in a CHO‐K1 clone that underwent transgene silencing during 10 weeks of continuous culture without selection (Figure 3A). To test for a causal relationship, we employed dCas9 fused to TET1, a 5‐methylcytosine dioxygenase that catalyzes demethylation at CpG dinucleotides (Figure 3B). Use of dCas9‐TET1 and sgRNAs targeting the CMV promoter fully reversed the high CpG methylation observed after 10 weeks in culture to levels seen when culture began (Figure 3A). This demethylation was accompanied by increased mAb mRNA levels (Figure 3C, D). This provides evidence that CpG methylation of the CMV promoter can directly inhibit transgene expression. However, the dCas9‐TET1 did not restore mRNA levels to those observed at the start of culture, even though the elevated DNA methylation was fully reversed. The data suggest that methylation of CMV promoter DNA contributes only partially to transgene silencing in this system.

**FIGURE 4:**
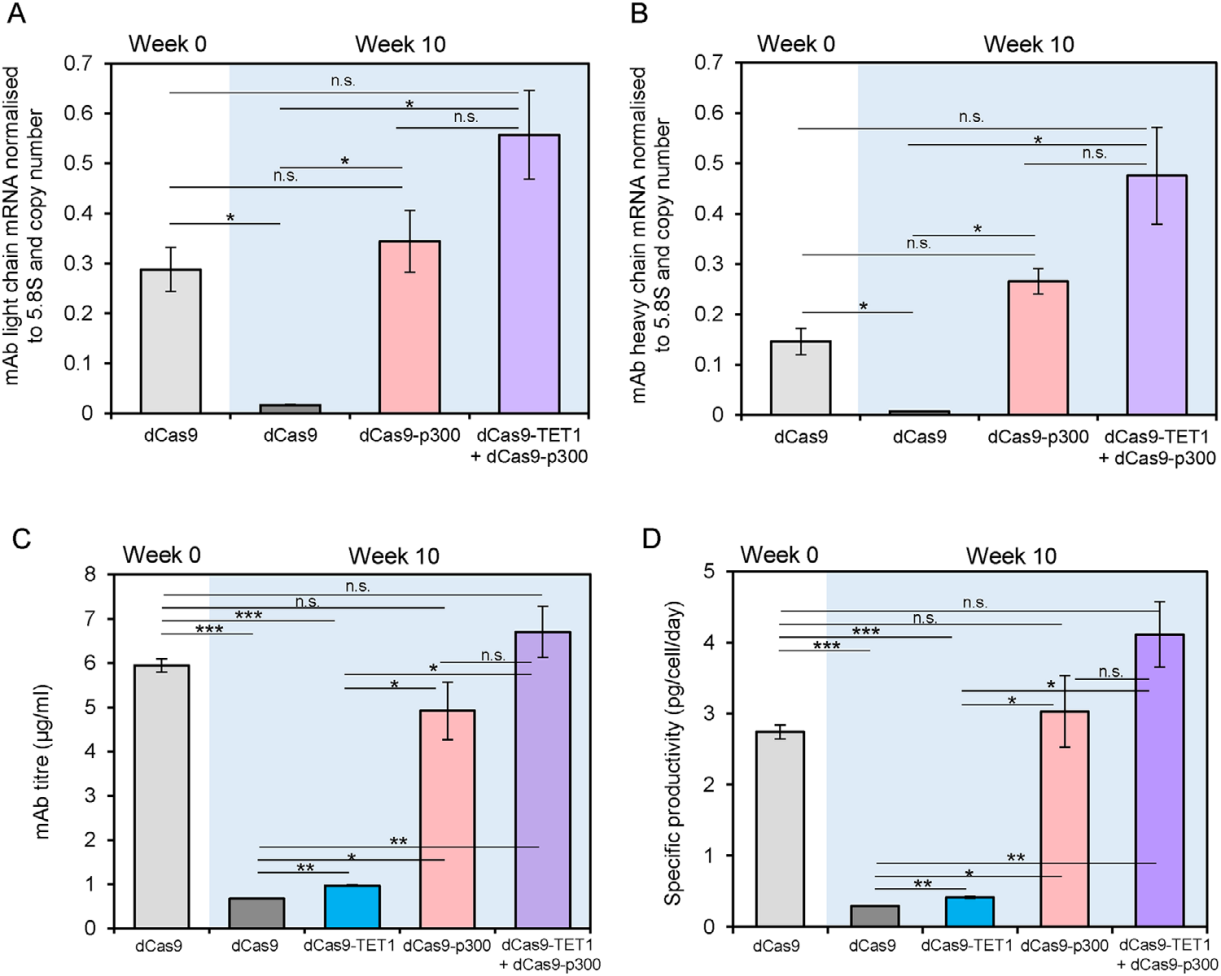
Histone acetylation has a greater influence on expression than CpG methylation at the CMV promoter. (A,B) mAb light chain (A) and heavy chain (B) mRNA expression levels in the same CHO‐K1 cell line measured at week 0 and week 10 of culture expressing either dCas9 alone, dCas9‐p300 or a combination of dCas9‐p300 and dCas9‐TET1. mRNA levels are normalized to 5.8S rRNA and copy number. (C) mAb titers of cell pools stably expressing dCas9 alone at weeks 0 and 10, dCas9‐TET1, dCas9‐p300 and both dCas9‐p300 and dCas9‐TET1 at week 10. (D) Cell‐specific productivities (q_p_s) are calculated using titer values. *p < 0.05, **p < 0.01, ***p < 0.001, n.s. not significant (*t*‐test). Error bars represent ± SEM, *n* = 3.

In contrast to the limited effect of dCas9‐TET1, the expression of dCas9‐p300 fully reversed the inhibition of mAb mRNAs observed after 10 weeks of continuous culture (Figure 4A,B). Induction by dCas9‐p300 is significantly stronger than that of dCas9‐TET1 (*p* < 0.05), despite the potency of dCas9‐TET1 in reversing promoter methylation. Combining these epigenetic regulators raised mAb mRNA levels still further, but the synergism did not reach statistical significance. The data suggest that histone acetylation influences transgene expression in this system more potently than the methylation of promoter DNA. These effects on mRNA levels are reflected by the titer of secreted mAb and specific productivity (Figure 4C,D).

### 3.5 Targeting the Catalytic Domain of p300 to the CMV Promoter Attracts BRD4 and Promotes the Elongation Phase of Transcription

Acetylation of nucleosomes at promoters has been associated with increasing access to general transcription factors, such as the TATA‐binding protein (TBP), by weakening the interactions between core histones and the DNA [13]. In this way, assembly of the pre‐initiation complex and recruitment of RNA polymerases are facilitated upon histone acetylation, leading to increased transcriptional output [44]. Acetylation of histones has also been shown to disrupt higher‐order folding of chromatin, leading to greater accessibility and transcription [45].

**FIGURE 5:**
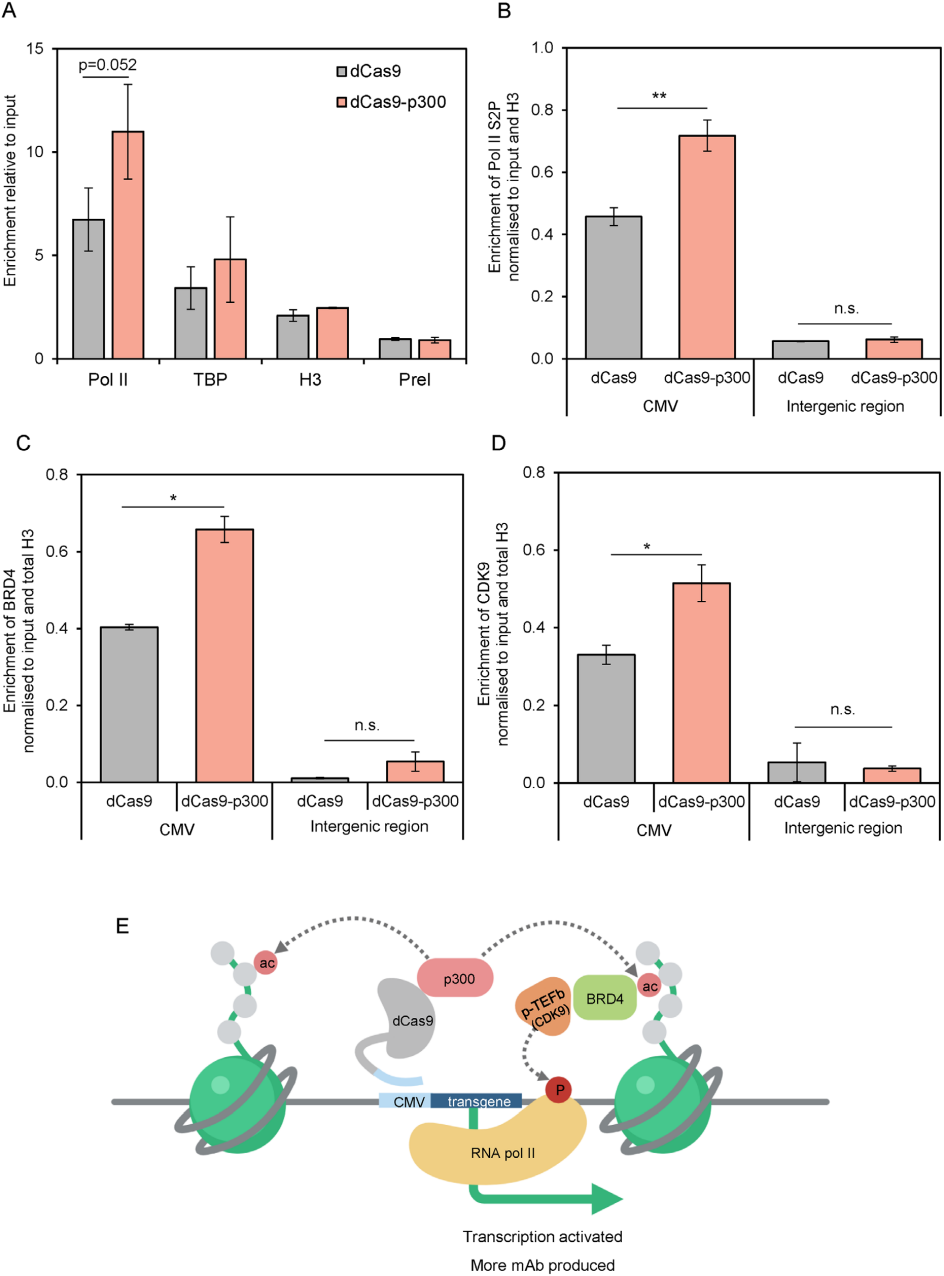
p300 increases expression by promoting the active form of RNA polymerase II. (A) ChIP data showing enrichment of total RNA polymerase II and TBP. Total histone H3 and preimmune serum (PreI) represent positive and negative controls respectively. Data is shown normalized to input. (B–D) ChIP data showing enrichment of RNA polymerase II S2P (B), BRD4 (C), and CDK9 (D) at the CMV promoter and an intergenic region. (E) Schematic representation of a proposed mechanism by which p300‐induced histone acetylation promotes transcription initiation and elongation via recognition of H3K27ac by the BRD4 bromodomain; BRD4 recruits p‐TEFb, which in turn activates RNA polymerase II by phosphorylation of its C‐terminal domain on the serine 2 residue. Data are shown normalized to input and total H3. *p < 0.05, **p < 0.01 (*t* test). Error bars represent ± SEM, *n* = 3.

To explore how a locus‐specific increase in H3K27ac at the CMV promoter gives rise to the increase in mAb mRNA (Figure 2D,F), enrichment of TBP and RNA polymerase II (Pol II) at the transcriptional start site was assessed in cells expressing dCas9 and dCas9‐p300. ChIP‐qPCR using anti‐TBP or anti‐Pol II antibodies was carried out with primers against the CMV promoter region around the transcription start site. Elevated binding of TBP (1.4‐fold) and Pol II (1.7‐fold) was observed in cells expressing dCas9‐p300 compared to unfused dCas9 (Figure 5A). However, these changes did not reach statistical significance (*p* > 0.05) and may be insufficient to explain the substantial increase in mRNA (Figure 2F). We therefore considered whether acetylation might also impact a step in the transcription cycle downstream from the recruitment of Pol II.

Acetylated histones can be recognized by proteins with bromodomains, which have important roles in transcription regulation [46]. A key example is BRD4 (bromodomain‐containing protein 4), which binds histones with acetylated lysines and recruits the positive transcription elongation factor P‐TEFb [46, 47]. Heptad repeat sequences in the C‐terminal domain of Pol II are phosphorylated by P‐TEFb on serine 2, which triggers promoter escape and stimulates transcript elongation [47]. In this way, acetylation of histones associated with promoters can stimulate transcription steps that follow Pol II recruitment, which are often rate‐limiting for output. To explore whether this mechanism influences the expression of mAb transgenes from the CMV promoter in CHO cells, ChIP‐qPCR was used to assess enrichment at the promoter and transgene of the activated form of Pol II, which is phosphorylated on serine 2 of its heptad repeats (Pol II S2P). Indeed, a significant increase (1.6‐fold, *p* < 0.01) in activated Pol II was detected at the CMV promoter when dCas9‐p300 was targeted, compared to dCas9 alone, an effect not seen at an untargeted intergenic region (Figure 5B). Serine 2 is phosphorylated by CDK9 (cyclin‐dependent kinase 9), the kinase subunit of the P‐TEFb complex, which is recruited by BRD4. Significant enrichment of both BRD4 (1.6‐fold, *p* < 0.05) and CDK9 (1.6‐fold, *p* < 0.05) was observed at the CMV promoter when targeted by dCas9‐p300, relative to the dCas9 negative control (Figure 5C,D). Comparable changes were not detected at the control intergenic site.

These data support a mechanism in which targeted recruitment of dCas9‐p300 to the CMV promoter stimulates acetylation of H3K27 to create a recognition site for BRD4. P‐TEFb is recruited by BRD4 and phosphorylates Pol II on serine 2, a modification that overcomes stalling and stimulates escape from the promoter and entry into the elongation phase of mRNA synthesis, leading to increased production of mRNA (Figure 5E). Raising the expression of the mAb mRNAs can lead to significant improvements in mAb titer and specific productivity.

## 4 Discussion

Despite its status as a powerful promoter and its frequent industrial application, our study found suboptimal acetylation of H3K27 at the CMV promoter in CHO cells. This may have a significant impact on transgene expression, as H3K27ac is strongly linked to transcriptional activity [12, 13, 14]. The deficit in H3K27ac can be remediated specifically using dCas9‐p300. The binding of BRD4 to H3K27ac allows the recruitment of PTEF‐b and subsequent phosphorylation of Pol II, which facilitates promoter escape and transcript elongation. Our data support a causal pathway linking locus‐specific acetylation at the CMV promoter to substantially increased transcription of mAb transgenes, ultimately elevating specific productivity in CHO cells. We suggest that promoter escape by Pol II into active transcript elongation may be a rate‐limiting step in transgene expression in this context, which can be alleviated by increasing the acetylation of histones at the CMV promoter. This offers the potential to raise production.

The weaker H3K27ac at the exogenous CMV promoter in transfected CHO cells compared to the endogenous GAPDH gene can be explained by the random integration of transgenes into the genome following transfection; it is unlikely that all copies integrate into euchromatic regions that support expression, as ∼90% of the genome is quiescent or repressed, with little or no transcription [48]. Specifically targeting the HAT domain of p300 to the CMV promoter suffices to increase H3K27ac and stimulate transgene expression substantially (Figure 2F–H). This suggests that the silencing of at least some promoter copies is reversible, a valuable discovery. Previous studies using HDAC inhibitors also implied reversibility, but indirect effects complicate their interpretation; for example, increased global acetylation might induce genes encoding transcription factors that regulate the transgene. Our targeted approach provides stronger evidence that acetylation localized to the CMV promoter is sufficient to raise transgene expression. We confirmed binding specificity, but the possibility remains of off‐target effects. However, these are unlikely to be extensive; indeed, only two off‐target sites for dCas9‐p300 were found by genome‐wide analysis in previous work [26, 28, 29].

We opted to use cell populations, as opposed to clonal cell lines, as the former presents the opportunity to test responses from a variety of insertion sites. The diversity of contexts was further increased by examining cell populations from three independent transfections of dCas9 fusion proteins. Comparing responses of the resultant stably‐expressing pools of cells allowed us to sample the conditions of multiple insertion sites within the populations.

Our findings demonstrate that increased titer and specific productivity of CHO cells can be achieved by targeted acetylation that raises the expression of transgene mRNA. The 1.8‐fold increase in product titer was far less substantial than the mRNA induction, but yield can be limited by bottlenecks in assembly, processing or secretion [49]. Nevertheless, an 80% boost in titer is a substantial improvement. Epigenetic control of the CMV promoter is likely to be similar in industrial systems to what we observed in CHO‐K1 cells, but this will need to be tested in each context.

## 5 Conclusion

Our results demonstrate that dCas9‐p300 can deliver robust, programmable and targeted induction of CMV promoter‐driven recombinant gene expression in CHO cells. The data support a causal relationship between directed histone acetylation and transgene activation via BRD4, pTEF‐b and phosphorylation of RNA polymerase II. This precise epigenetic engineering could combat silencing in long‐term culture. Furthermore, there is potential to combine dCas9‐p300 with light‐inducible or chemically‐inducible control to allow dynamic regulation [36, 50]. Targeted epigenetic editing has the potential to improve specific productivity without altering DNA sequences, restricting integration sites or introducing wide and unpredictable effects on the CHO epigenome.

## Author Contributions

**Sienna P. Butterfield**: conceptualization, methodology, verification, formal analysis, investigation, writing–original draft, visualization. **Rebecca E. Sizer**: methodology, investigation, formal analysis, writing–review and editing. **Fay L. Saunders**: supervision, writing–review and editing. **Robert J. White**: conceptualization, writing–review and editing, supervision, project administration, funding acquisition.

## Conflicts of Interest

The authors declare no conflicts of interest.

## Data Availability

The data that support the findings of this study are available from the corresponding author upon reasonable request.

## References

[1] G. Walsh , “Biopharmaceutical Benchmarks 2018,” Nature Biotechnology 36, no. 12 (2018): 1136–1145. 10.1038/nbt.4305 30520869

[2] G. Walsh and E. Walsh , “Biopharmaceutical Benchmarks 2022,” Nature Biotechnology 40, no. 12 (2022): 1722–1760. 10.1038/s41587-022-01582-x PMC9735008 36471135

[3] F. Nematpour , F. Mahboudi , and B. Vaziri , “Evaluating the Expression Profile and Stability of Different UCOE Containing Vector Combinations in mAb‐Producing CHO Cells,” BMC Biotechnology 17, no. 1 (2017): 18. 28228095 10.1186/s12896-017-0330-0 PMC5322649

[4] H. Dahodwala and K. H. Lee , “The Fickle CHO: A Review of the Causes, Implications, and Potential Alleviation of the CHO Cell Line Instability Problem,” Current Opinion in Biotechnology 60 (2019): 128–137. 30826670 10.1016/j.copbio.2019.01.011

[5] H. Dhiman , M. Campbell , M. Melcher , K. D. Smith , and N. Borth , “Predicting Favorable Landing Pads for Targeted Integrations in Chinese Hamster Ovary Cell Lines by Learning Stability Characteristics From Random Transgene Integrations,” Computational and Structural Biotechnology Journal 18 (2020): 3632–3648. 33304461 10.1016/j.csbj.2020.11.008 PMC7710658

[6] K. Strutzenberger , N. Borth , R. Kunert , W. Steinfellner , and H. Katinger , “Changes During Subclone Development and Ageing of Human Antibody‐Producing Recombinant CHO Cells,” Journal of Biotechnology 69, no. 2–3 (1999): 215–226. 10361728 10.1016/s0168-1656(99)00044-9

[7] Y. Yang , C. Mariati , and M. G. S. Yap , “DNA Methylation Contributes to Loss in Productivity of Monoclonal Antibody‐Producing CHO Cell Lines,” Journal of Biotechnology 147, no. 3–4 (2010): 180–185. 20430058 10.1016/j.jbiotec.2010.04.004

[8] M. J. Pikaart , F. Recillas‐Targa , and G. Felsenfeld , “Loss of Transcriptional Activity of a Transgene Is Accompanied by DNA Methylation and Histone Deacetylation and Is Prevented by Insulators,” Genes & Development 12, no. 18 (1998): 2852–2862. 9744862 10.1101/gad.12.18.2852 PMC317165

[9] N. Veith , H. Ziehr , R. A. F. MacLeod , and S. M. Reamon‐Buettner , “Mechanisms Underlying Epigenetic and Transcriptional Heterogeneity in Chinese Hamster Ovary (CHO) Cell Lines,” BMC Biotechnology 16 (2016): 6. 26800878 10.1186/s12896-016-0238-0 PMC4722726

[10] V. Paredes , J. S. Park , Y. Jeong , J. Yoon , and K. Baek , “Unstable Expression of Recombinant Antibody During Long‐Term Culture of CHO Cells Is Accompanied by Histone H3 Hypoacetylation,” Biotechnology Letters 35, no. 7 (2013): 987–993. 23468139 10.1007/s10529-013-1168-8

[11] A. Wippermann , O. Rupp , K. Brinkrolf , R. Hoffrogge , and T. Noll , “Integrative Analysis of DNA Methylation and Gene Expression in Butyrate‐Treated CHO Cells,” Journal of Biotechnology 257 (2017): 150–161. 27890772 10.1016/j.jbiotec.2016.11.020

[12] B. M. Turner , “Defining an Epigenetic Code,” Nature Cell Biology 9, no. 1 (2007): 2–6. 17199124 10.1038/ncb0107-2

[13] D. Y. Lee , J. J. Hayes , D. Pruss , and A. P. Wolffe , “A Positive Role for Histone Acetylation in Transcription Factor Access to Nucleosomal DNA,” Cell 72, no. 1 (1993): 73–84. 8422685 10.1016/0092-8674(93)90051-q

[14] S. L. Berger , “The Complex Language of Chromatin Regulation During Transcription,” Nature 447, no. 7143 (2007): 407–412. 17522673 10.1038/nature05915

[15] J. Feichtinger , I. Hernández , and C. Fischer , “Comprehensive Genome and Epigenome Characterization of CHO Cells in Response to Evolutionary Pressures and Over Time,” Biotechnology and Bioengineering 113, no. 10 (2016): 2241–2253. 27072894 10.1002/bit.25990 PMC5006888

[16] T. Tharmalingam , H. Barkhordarian , and N. Tejeda , “Characterization of Phenotypic and Genotypic Diversity in Subclones Derived From a Clonal Cell Line,” Biotechnology Progress 34, no. 3 (2018): 613–623. 29882350 10.1002/btpr.2666 PMC6099272

[17] N. Marx , P. Eisenhut , M. Weinguny , G. Klanert , and N. Borth , “How to Train Your Cell—Towards Controlling Phenotypes by Harnessing the Epigenome of Chinese Hamster Ovary Production Cell Lines,” Biotechnology Advances 56 (2022): 107924. 35149147 10.1016/j.biotechadv.2022.107924

[18] A. Wright , A. Semyonov , and G. Dawes , “Diverse Plasmid DNA Vectors by Directed Molecular Evolution of Cytomegalovirus Promoters,” Human Gene Therapy 16, no. 7 (2005): 881–892. 16000069 10.1089/hum.2005.16.881

[19] L. A. Bailey , D. Hatton , R. Field , and A. J. Dickson , “Determination of Chinese Hamster Ovary Cell Line Stability and Recombinant Antibody Expression During Long‐Term Culture,” Biotechnology and Bioengineering 109, no. 8 (2012): 2093–2103. 22896849 10.1002/bit.24485

[20] A. J. Brown , B. Sweeney , D. O. Mainwaring , and D. C. James , “NF‐κB, CRE and YY1 Elements Are Key Functional Regulators of CMV Promoter‐Driven Transient Gene Expression in CHO Cells,” Biotechnology Journal 10, no. 7 (2015): 1019–1028. 25612069 10.1002/biot.201400744

[21] A. Osterlehner , S. Simmeth , and U. Göpfert , “Promoter Methylation and Transgene Copy Numbers Predict Unstable Protein Production in Recombinant Chinese Hamster Ovary Cell Lines,” Biotechnology and Bioengineering 108, no. 11 (2011): 2670–2681. 21618470 10.1002/bit.23216

[22] S. Spencer , A. Gugliotta , J. Koenitzer , H. Hauser , and D. Wirth , “Stability of Single Copy Transgene Expression in CHOK1 Cells Is Affected by Histone Modifications but Not by DNA Methylation,” Journal of Biotechnology 195 (2015): 15–29. 25533398 10.1016/j.jbiotec.2014.12.009

[23] A. J. Brown , B. Sweeney , D. O. Mainwaring , and D. C. James , “Synthetic Promoters for CHO Cell Engineering,” Biotechnology and Bioengineering 111, no. 8 (2014): 1638–1647. 24615264 10.1002/bit.25227

[24] Y. B. Johari , J. M. Scarrott , and T. H. Pohle , “Engineering of the CMV Promoter for Controlled Expression of Recombinant Genes in HEK293 Cells,” Biotechnology Journal 17, no. 8 (2022): e2200062. 35482470 10.1002/biot.202200062

[25] B. Moritz , L. Woltering , P. B. Becker , and U. Göpfert , “High Levels of Histone H3 Acetylation at the CMV Promoter Are Predictive of Stable Expression in Chinese Hamster Ovary Cells,” Biotechnology Progress 32, no. 3 (2016): 776–786. 27073121 10.1002/btpr.2271

[26] F. Saunders , B. Sweeney , M. N. Antoniou , P. Stephens , and K. Cain , “Chromatin Function Modifying Elements in an Industrial Antibody Production Platform—Comparison of UCOE, MAR, STAR and cHS4 Elements,” PLoS ONE 10, no. 4 (2015): e0120096. 25849659 10.1371/journal.pone.0120096 PMC4388700

[27] F. L. Saunders (2011). An investigation into the role of chromatin modifying elements on the production of recombinant antibodies from CHO Cells [Phd, The Open University], 10.21954/ou.ro.0000ed85.

[28] R. E. Sizer , N. Chahid , S. P. Butterfield , D. Donze , N. J. Bryant , and R. J. White , “TFIIIC‐Based Chromatin Insulators Through Eukaryotic Evolution,” Gene 835 (2022): 146533. 35623477 10.1016/j.gene.2022.146533

[29] R. E. Sizer and R. J. White , “Use of Ubiquitous Chromatin Opening Elements (UCOE) as Tools to Maintain Transgene Expression in Biotechnology,” Computational and Structural Biotechnology Journal 21 (2023): 275–283. 36582439 10.1016/j.csbj.2022.11.059 PMC9764128

[30] Z. Betts and A. J. Dickson , “Ubiquitous Chromatin Opening Elements (UCOEs) Effect on Transgene Position and Expression Stability in CHO Cells Following Methotrexate (MTX) Amplification,” Biotechnology Journal 11, no. 4 (2016): 554–564. 26632501 10.1002/biot.201500159

[31] D. Kim , C. Yoon , and G. M. Lee , “Small Molecule Epigenetic Modulators for Enhancing Recombinant Antibody Production in CHO Cell Cultures,” Biotechnology and Bioengineering 119, no. 3 (2022): 820–831. 34961935 10.1002/bit.28013

[32] R. Lillico , M. G. Sobral , N. Stesco , et al., “HDAC Inhibitors Induce Global Changes in Histone Lysine and Arginine Methylation and Alter Expression of Lysine Demethylases,” Journal of Proteomics 133 (2016): 125–133. 26721445 10.1016/j.jprot.2015.12.018

[33] L. S. Qi , M. H. Larson , L. A. Gilbert , et al., “Repurposing CRISPR as an RNA‐Guided Platform for Sequence‐Specific Control of Gene Expression,” Cell 184, no. 3 (2021): 844. 33545038 10.1016/j.cell.2021.01.019

[34] H. O'Geen , C. Ren , C. M. Nicolet , et al., “dCas9‐Based Epigenome Editing Suggests Acquisition of Histone Methylation Is Not Sufficient for Target Gene Repression,” Nucleic Acids Research 45, no. 17 (2017): 9901–9916. 28973434 10.1093/nar/gkx578 PMC5622328

[35] H. O'Geen , S. L. Bates , S. S. Carter , et al., “Ezh2‐dCas9 and KRAB‐dCas9 Enable Engineering of Epigenetic Memory in a Context‐Dependent Manner,” Epigenetics & Chromatin 12, no. 1 (2019): 26. 31053162 10.1186/s13072-019-0275-8 PMC6498470

[36] I. B. Hilton , A. M. D'Ippolito , C. M. Vockley , et al., “Epigenome Editing by a CRISPR‐Cas9‐Based Acetyltransferase Activates Genes From Promoters and Enhancers,” Nature Biotechnology 33, no. 5 (2015): 510–517. 10.1038/nbt.3199 PMC4430400 25849900

[37] N. Marx , H. Dhiman , V. Schmieder , et al., “Enhanced Targeted DNA Methylation of the CMV and Endogenous Promoters With dCas9‐DNMT3A3L Entails Distinct Subsequent Histone Modification Changes in CHO Cells,” Metabolic Engineering 66 (2021): 268–282. 33965614 10.1016/j.ymben.2021.04.014

[38] N. Marx , C. Grünwald‐Gruber , N. Bydlinski , et al., “CRISPR‐Based Targeted Epigenetic Editing Enables Gene Expression Modulation of the Silenced Beta‐Galactoside Alpha‐2,6‐Sialyltransferase 1 in CHO Cells,” Biotechnology Journal 13, no. 10 (2018): e1700217. 29802757 10.1002/biot.201700217

[39] T. H. J. Kwaks , R. G. A. B. Sewalt , R. van Blokland , et al., “Targeting of a Histone Acetyltransferase Domain to a Promoter Enhances Protein Expression Levels in Mammalian Cells,” Journal of Biotechnology 115, no. 1 (2005): 35–46. 15607223 10.1016/j.jbiotec.2004.07.012

[40] D. E. Bauer , M. C. Canver , and S. H. Orkin , “Generation of Genomic Deletions in Mammalian Cell Lines via CRISPR/Cas9,” Journal of Visualized Experiments: JoVE 95 (2015): e52118. 10.3791/52118 PMC4279820 25549070

[41] S. Ghisletti , I. Barozzi , F. Mietton , et al., “Identification and Characterization of Enhancers Controlling the Inflammatory Gene Expression Program in Macrophages,” Immunity 32, no. 3 (2010): 317–328. 20206554 10.1016/j.immuni.2010.02.008

[42] T. Aki , S. Yanagisawa , and H. Akanuma , “Identification and Characterization of Positive Regulatory Elements in the Human Glyceraldehyde 3‐phosphate Dehydrogenase Gene Promoter,” Journal of Biochemistry 122, no. 2 (1997): 271–278. 9378702 10.1093/oxfordjournals.jbchem.a021749

[43] B. T. Weinert , T. Narita , S. Satpathy , et al., “Time‐Resolved Analysis Reveals Rapid Dynamics and Broad Scope of the CBP/p300 Acetylome,” Cell 174, no. 1 (2018): 231–244.e12. 29804834 10.1016/j.cell.2018.04.033 PMC6078418

[44] C. N. J. Ravarani , T. Flock , S. Chavali , M. Anandapadamanaban , M. M. Babu , and S. Balaji , “Molecular Determinants Underlying Functional Innovations of TBP and Their Impact on Transcription Initiation,” Nature Communications 11, no. 1 (2020): 2384. 10.1038/s41467-020-16182-z PMC7221094 32404905

[45] C. Tse , T. Sera , A. P. Wolffe , and J. C. Hansen , “Disruption of Higher‐Order Folding by Core Histone Acetylation Dramatically Enhances Transcription of Nucleosomal Arrays by RNA Polymerase III,” Molecular and Cellular Biology 18, no. 8 (1998): 4629–4638. 9671473 10.1128/mcb.18.8.4629 PMC109049

[46] G. A. Josling , S. A. Selvarajah , M. Petter , and M. F. Duffy , “The Role of Bromodomain Proteins in Regulating Gene Expression,” Genes 3, no. 2 (2012): 320–343. 24704920 10.3390/genes3020320 PMC3899951

[47] M. K. Jang , K. Mochizuki , M. Zhou , H.‐S. Jeong , J. N. Brady , and K. Ozato , “The Bromodomain Protein Brd4 is a Positive Regulatory Component of P‐TEFb and Stimulates RNA Polymerase II‐Dependent Transcription,” Molecular Cell 19, no. 4 (2005): 523–534. 16109376 10.1016/j.molcel.2005.06.027

[48] A. C. Gasser , “Chromosomes Expression Mechanisms: New Excitement Over Old Word: ‘Chromatin’,” Current Opinion in Genetics & Development 8 (1998): 137–139. 9664062 10.1016/s0959-437x(98)80133-0

[49] M. Mason , B. Sweeney , K. Cain , P. Stephens , and S. T. Sharfstein , “Identifying Bottlenecks in Transient and Stable Production of Recombinant Monoclonal‐Antibody Sequence Variants in Chinese Hamster Ovary Cells,” Biotechnology Progress 28, no. 3 (2012): 846–855. 22467228 10.1002/btpr.1542 PMC3394691

[50] L. R. Polstein and C. A. Gersbach , “A Light‐Inducible CRISPR‐Cas9 System for Control of Endogenous Gene Activation,” Nature Chemical Biology 11, no. 3 (2015): 198–200. 25664691 10.1038/nchembio.1753 PMC4412021

